# Repeated lower extremities hot water bathing (REHAB), a rehabilitative strategy in Parkinson's disease (PD) patients with sleep disorders: rationale, design and protocol for a randomized controlled study

**DOI:** 10.3389/fneur.2026.1753102

**Published:** 2026-03-24

**Authors:** Xuemei Wang, Yuchuan Ding, Omar Elmadhoun, Yuan Yuan, Yanna Tong, Lipeng Cai, Qiling Ji, Yanling Wang, Fengwu Li, Xiaokun Geng

**Affiliations:** 1 Department of Neurology, Beijing Luhe Hospital, Capital Medical University, Beijing, China; 2 Department of Neurosurgery, Wayne State University School of Medicine, Detroit, MI, United States; 3 Division of Critical Care, Department of Anesthesiology & Perioperative Medicine, Mayo Clinic, Rochester, NJ, United States

**Keywords:** aerobic exercise, neurodegenerative disease, non-motor symptoms, Parkinson's disease sleep scale (PDSS), physical therapies, polysomnography (PSG)

## Abstract

**Background:**

Sleep disturbances are common non-motor symptoms in Parkinson's disease (PD), significantly diminishing patients' quality of life. Management typically involves sleep hygiene education, psychotherapy, pharmacological and non-pharmacological interventions. This study aims to explore the safety and effectiveness of repeated lower extremity hot water bathing (REHAB) as a potential treatment for sleep disorders in this patient population.

**Methods:**

This is a randomized, controlled, open-label with trial outcome assessment. A total of 40 elderly patients with advanced PD will be enrolled and randomly assigned (1:1) to either a REHAB group or a control group. The control group will receive guideline-based usual care. While the REHAB group will receive the same usual care plus 30-min REHAB sessions, conducted daily for 5–7 days per week over 4 weeks, followed by a 2-week observation period. The primary objective of this study is to evaluate the safety of REHAB in elderly patients with advanced PD. These safety concerns include orthostatic hypotension associated with autonomic dysfunction, scalds or intolerance to hot water bathing due to sensory disorders (both common in PD patients), and signs of tissue damage. Secondary objectives include evaluating the impact of REHAB on sleep quality using the Parkinson's Disease Sleep Scale (PDSS) and polysomnography (PSG) at weeks 0, 4, and 6.

**Discussion:**

This novel randomized controlled trial will assess the safety and potential therapeutic benefits of REHAB for sleep disorders in elderly patients with advanced PD. The findings will inform design considerations for future phase III studies and may introduce a new rehabilitation strategy for managing sleep disturbances in this population.

**Trial registration:**

http://www.chictr.org.cn/, identifier: ChiCTR2400092087.

## 1 Introduction

Parkinson's disease (PD) is a prevalent neurodegenerative disease marked by both motor and non-motor symptoms (1), with sleep disturbances affecting up to 98% of patients (2). The manifestations of PD sleep disturbances include sleep fragmentation, insomnia, rapid eye movement sleep behavior disorder (RBD), restless legs syndrome (RLS), and excessive daytime sleepiness (EDS). Such symptoms exacerbate motor dysfunction and reduce quality of life, particularly in advanced stages (3).

The management of sleep disorders in patients with PD often requires a collaborative and multifaceted approach, typically beginning with sleep hygiene education and effective non-pharmacological treatments. Consequently, non-pharmacological interventions, such as psychotherapy and physical therapies, are increasingly recommended as first-line treatments (4). Physical therapies, including exercise (5, 6), light therapy (7), transcranial magnetic stimulation (TMS) (8), and deep brain stimulation (DBS) (9), have demonstrated benefits for sleep quality in PD patients. However, their widespread use is limited by factors such as impaired mobility, the need for specialized equipment and trained professionals, and economic considerations.

The mechanisms of sleep disorders in PD are complex, involving neuronal degeneration and the accumulation of α-synuclein and tau proteins in brain regions critical for sleep regulation (10). Heat therapy has been proposed as a potential therapeutic approach by promoting the delivery of molecular mediators within extracellular vesicles, which help maintain balanced neurotransmission (11). Pre-clinical studies have shown that exposure to high temperatures can elevate circulating levels of dopamine and glutamate (12), potentially modulating dopaminergic transmission and receptor sensitivity while increasing striatal activity. In addition, heat therapy may upregulate heat shock proteins (HSPs), which play a protective role against α-synuclein aggregation in PD. Given their role in protein homeostasis, HSP expression has been proposed as a therapeutic target for neurodegenerative diseases (13).

Another area of focus is inflammation, as early stages of PD have been linked to gastrointestinal inflammation that may spread to the central nervous system (CNS) via the vagus nerve (14). Heat therapy has been shown to modulate inflammatory pathways, reduce chronic low-grade inflammation, induce anti-apoptotic and antioxidative effects, and enhance mitochondrial respiratory capacity. Collectively, these effects may slow neurodegenerative progression (15). Evidence suggests that heat therapy, through methods like hot water bathing or sauna use, may serve as an effective alternative to aerobic exercise for individuals with mobility impairments, offering potential benefits for the aging brain (16, 17). Regular heat therapy has been associated with a reduced risk of developing neurodegenerative conditions (18).

Thermoregulatory mechanisms play a key role in sleep, because warming is directly linked to sleep initiation. Elevating skin temperature can stimulate warm-sensitive neurons in the pre-optic area and anterior hypothalamus, increasing their firing rate and promoting sleep onset (19). Early studies have demonstrated that manipulating ambient temperature enhances sleep depth in mice (20). In both humans and other mammals, direct skin warming has been shown to shorten sleep latency, increase slow-wave-sleep (SWS), enhance Non-Rapid Eye Movement sleep (NREM) consolidation, and decrease Rapid Eye Movement sleep (REM) (21). In elderly individuals with insomnia, warming distal skin temperature has been shown to enhance slow-wave sleep (22). Thermal therapy applied to the extremities has also been explored as a complementary treatment to improve sleep quality in patients with chronic heart failure (23). Heat therapy has demonstrated potential in alleviating sleep disturbances following traumatic brain injury (24), promoting relaxation, reducing pain perception, and discomfort during sleep in cancer patients (25), decreasing symptoms of restless leg syndrome during pregnancy (26, 27), and improving sleep quality in chronic heart failure patients (23). Notably, the 2019 Global Sauna Cross-sectional Survey reported benefits of sauna use for health, particularly in terms of mental health and sleep, with relatively few side effects (18).

The current trial aims to provide evidence regarding the safety and efficacy of heat therapy in elderly patients with advanced PD with sleep disorders. Hot bathing has been found to be safe and effective in young, healthy individuals, as well as in obese populations (28). However, there are occasional reports of hot bathing causing transient symptomatic hypotension (29) in some individuals. Patients with PD often experience autonomic nervous system dysfunction, which increases the risk of neurogenic orthostatic hypotension (30).

Therefore, the objective of this trial is to first assess the safety and then evaluate the clinical efficacy of repeated lower extremity hot water bathing (REHAB) on sleep in patients with PD. REHAB is a structured, multidisciplinary rehabilitation program that involves daily immersion of the lower extremities in warm water. In this study, participants in the intervention group will immerse their legs up to around mid-calf in 41 °C water for 30 min per session using a thermostatic footbath device (Changhong, model CDN-ZY8808C; dimensions: 44 × 41.5 × 36.5 cm; compliant with Chinese executive standards GB4706.1-2005 and GB4706.10-2008). The results of this study will provide valuable evidence for a home-based treatment for PD patients and their families.

## 2 Methods

### 2.1 Study design

This is a single-center, prospective, randomized controlled trial designed to enroll elderly patients with advanced PD. All participants will be informed of the clinical research and the requirements for giving informed consent. The research plan and informed consent will be approved by the regional ethics committee; the trial is registered at www.chictr.org.cn (Registration number: ChiCTR2400092087).

Forty PD patients will be randomly assigned in a 1:1 ratio to the REHAB group or the control group. The control group will receive daily medication and rehabilitation therapy as recommended by the International Movement Disorders Society (31). The intervention group will receive REHAB in addition to guideline-based usual care. An independent physician will monitor the participants' health and safety via WeChat video calls.

### 2.2 Inclusion and exclusion criteria

Participants will be recruited from both outpatient and inpatient centers at Beijing Luhe Hospital, Capital Medical University. The inclusion criteria are as follows: (1) diagnosis of PD according to the MDS clinical criteria; (2) 2.5 ≤ Hoehn-Yahr ≤ 4.0; (3) age between 60 and 80 years old; (4) PDSS score < 90; (5) stable condition and levodopa equivalent daily dose for ≥30 days; (6) obtained written informed consent from the participants or their legally authorized representative.

Exclusion criteria include patients diagnosed with mental illness (bipolar disorder, mania, schizophrenia etc.), substance abuse, uncontrolled cardiovascular disease, or other conditions such as local skin rupture, soft tissue injury, fracture, infection, and peripheral neuropathy in both lower extremities, that might impede their ability to participate in REHAB.

### 2.3 Randomization and blinding

This trial employs a randomized controlled, open-label, outcome assessor-blinded design (32).

Eligible participants will be allocated via a centralized randomization system. An independent statistician, not involved in participant recruitment, intervention delivery, or outcome assessment, will generate the random allocation sequence using the PROC PLAN procedure in SAS 9.4 software (SAS Institute Inc., Cary, NC, USA). A block randomization method will be used to ensure balanced group sizes throughout the recruitment period. The generated sequence will be securely stored within an independent central randomization system. Upon completion of screening and provision of informed consent, the study coordinator will access this central system to obtain the participant's unique group assignment (REHAB group or control group).

### 2.4 Interventions

#### 2.4.1 Control group

Patients in the control group will continue their usual care, which includes a stable dose of medication and daily exercise for the management of PD, as per the recommendations of the International Parkinson and Movement Disorder Society (33, 34).

#### 2.4.2 Intervention group (REHAB group)

**Figure 1:**
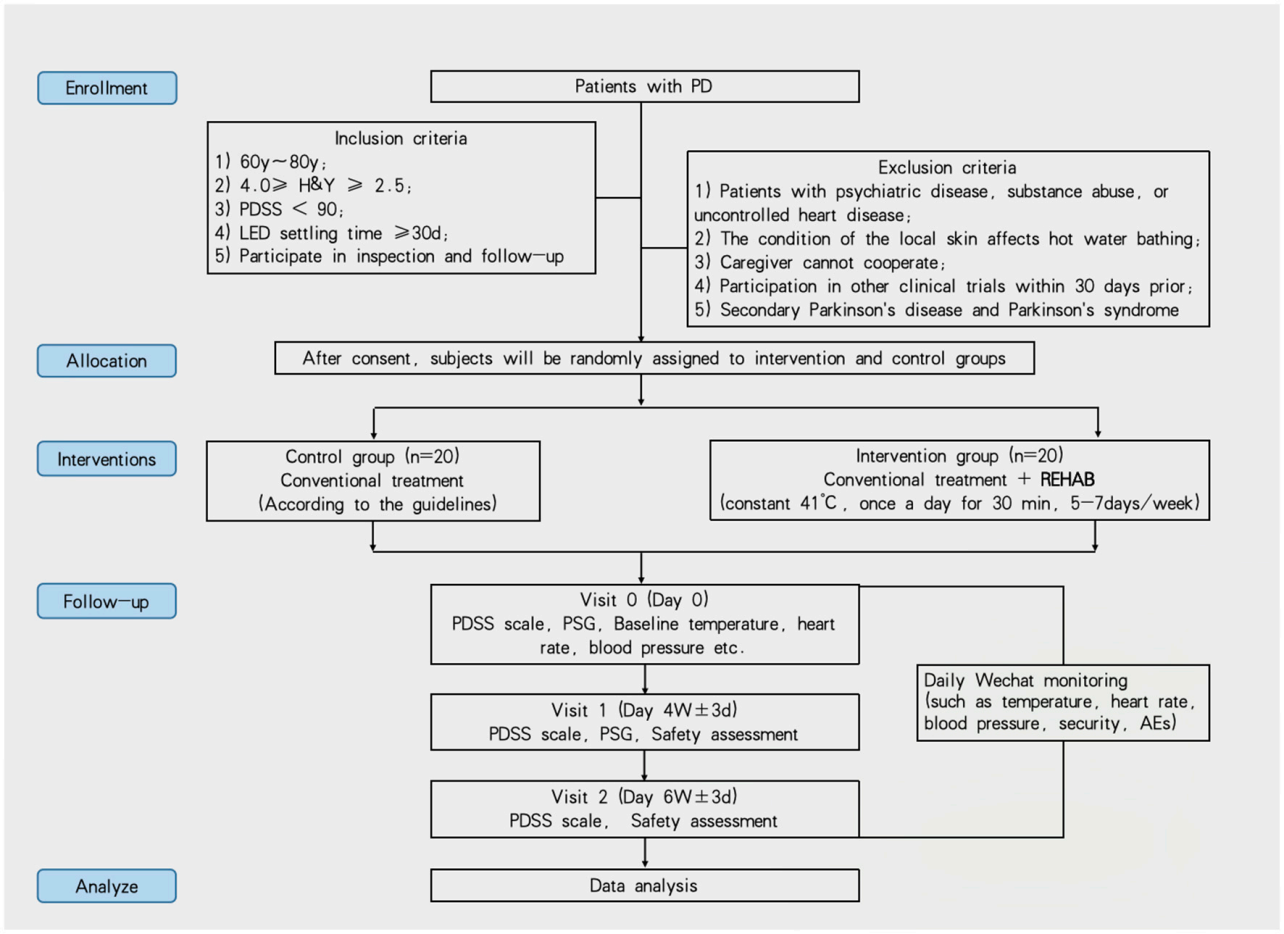
Flow chart of the repeated lower extremities hot water bathing study.

In addition to continuing their usual stable dose of medication and daily exercise, participants in the REHAB group will perform REHAB using a thermostatic footbath device (Changhong, CDN-ZY8808C, executive standard GB4706. 1-2005 GB4706. 10-2008. dimensions: 44 × 41.5 × 36.5 cm). Initially, the principal investigator will demonstrate the correct use of the footbath device to the patients. Participants will then immerse their lower extremities in 41 °C water for 30 min per session (24). This will be performed once daily, for 5–7 days per week, over a total period of 4 weeks (35). The recommended time for REHAB is 1 to 2 h prior to bedtime (Figure 1).

#### 2.4.3 Data collection and monitoring for all participants

Patients in both groups must maintain a daily log tracking their blood pressure, heart rate, and body temperature. For the intervention group, these measurements will be taken immediately before and after each REHAB session. All logs, which will also note any discomfort experienced, will be submitted to the researchers via WeChat. To ensure compliance with the study protocol, a study assistant will conduct daily check-ins with all participants over WeChat video.

Our study prioritizes the tolerability and safety of REHAB for patients. Exposure to 41 °C hot water bathing is generally considered safe, even during pregnancy (27). However, PD-associated autonomic dysfunction (e.g., hyperhidrosis, anhidrosis, orthostatic hypotension) may cause discomfort. To mitigate risks, participants will be advised to drink plenty of water, wipe away sweat with a towel, and lie flat under a thin blanket for 15 min after soaking to allow blood pressure to return to normal and reduce the potential risk of syncope-related falls. If participants experience nausea, dizziness, or significant discomfort, the treatment will be discontinued.

### 2.5 Assessments

The Parkinson's Disease Sleep Scale (PDSS) (36) will be used to assess the common sleep disturbances experienced by PD individuals. This 15-item self-report questionnaire covers six domains of nocturnal sleep issues and is widely used in clinical practice to quantify sleep disturbance severity in PD patients. Participants with PDSS scores under 90 will be included in the study. Assessments will occur at baseline (on the day of enrollment) and at weeks 4, 6 post-enrollment. Blinded investigators will administer assessments.

**Figure 2:**
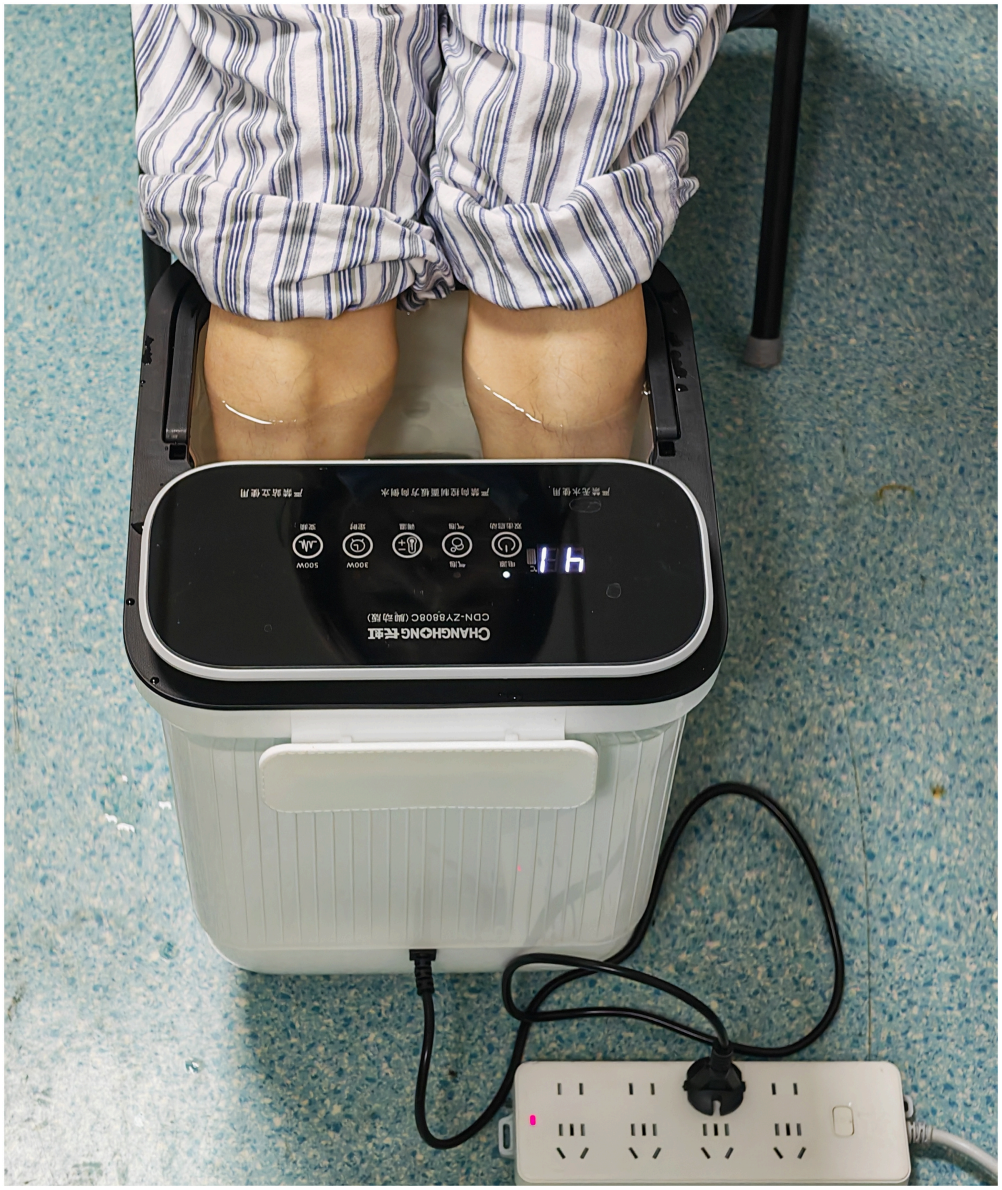
Diagram of lower leg and foot hot water bathing.

Polysomnography (PSG, Compumedics, AustraliaGermany, Type Grael) will be used to evaluate objective sleep parameters (2) before and after treatment. Total sleep time (TST), sleep efficiency (SE), sleep latency (SL), arousal index (AI), and non-rapid eye movement sleep stages (N1, N2, N3) will be recorded. To monitor safety and tolerability, adverse events (AEs) will be tracked throughout the study (Table 1; Figure 2).

**Table 1:** Baseline demographic and clinical data of the study participants.

**Variable**	**Treatment period**		**Follow-up period**
**Number of Visit**	**Visit 0 (Baseline)**	**Visit 1**	**Visit 2**
Time	0D	4W ± 3D	6W ± 3D
**Sociodemographic characteristics**			
Gender (M/F)	X		
Age (*y*)	X		
Duration of PD (*y*)	X		
Hoehn–Yahr (stage)	X		
UPDRSIII (“on” stage, score)	X	X	X
PDSS (score)	X	X	X
**PSG (Compumedics, Australia Germany, Type Grael)**			
Total sleep time (TST) (s)	X	X	
Sleep efficiency (SE) (%)	X	X	
Sleep latency (SL) (s)	X	X	
Arousal index (AI) (*n*)	X	X	
Non-rapid eye movement sleep stages N1 (%)	X	X	
Non-rapid eye movement sleep stages N2 (%)	X	X	
Non-rapid eye movement sleep stages N3 (%)	X	X	
Adverse events (AEs; *n*)		X^a^	X^b^
Adherence rate (%)		X^c^	X^d^

^a^Intervention monitoring data (V0 to Visit 1, Weeks 1–4): summarized from daily recordings. Adverse events: total count during the entire intervention period;

^b^Data from V1 to Visit 2 (Weeks 5–6);

^c^Adherence rate from V0 to V1: calculated as (number of days the participant followed the investigator's instructions/total expected days) × 100%;

^d^Adherence rate from V1 to V2: calculated similarly.

### 2.6 Outcomes

#### 2.6.1 Primary outcomes

The primary objective of this study is to assess the safety of REHAB. Adverse events will be closely monitored including: 1) symptomatic hypotension, which has been reported in association with hot bathing and may be exacerbated in PD due to autonomic dysfunction (29, 30); 2) Scald or intolerance as sensory impairments in PD may also lead to altered sensitivity to water temperature, increasing the risk of burns are intolerance to REHAB (37); 3) Systemic symptoms, such as headache, dizziness, palpitations, sweating, etc.; 4) Signs of tissue or neurovascular injury due to REHAB, including erythema, skin damage, tenderness upon palpation, and local edema.

All adverse events will be carefully assessed by trained research personnel blinded to randomization. Blood pressure, heart rate, and core body temperature we will be recorded before and after each session to further evaluate safety, and these physiological parameters will be incorporated into the safety analysis.

#### 2.6.2 Secondary outcomes

The secondary objective of this study is to evaluate the clinical effectiveness of REHAB by examining changes in PDSS scores and objective sleep measures (TST, SE, SL, AI, N1, N2, N3) from baseline to weeks 4 and 6 post-treatment. The study will evaluate improvements and in PDSS scores and compare PSG measures between participants receiving REHAB and those in the control group. The impact of REHAB will be determined by comparing pre- and post-intervention scores within the intervention group and between the intervention and control groups.

### 2.7 Sample size estimation

Due to the lack of prior clinical data on REHAB in this population, the study will serve as a foundational reference for future research. Prior literature (38) suggests that a sample side of 10–20 patients per group is sufficient for pilot studies. Dobkin et al. (39) note that most Phase I clinical rehabilitation pilot studies often begin with 6–12 participants. This study aims to enroll 20 patients in each group. The findings will inform safety and feasibility assessments while also guiding sample size estimations and power calculations for future Phase III trials.

### 2.8 Statistical analysis

Analysis will be performed on a per-protocol basis, including data only from participants who completed the full study protocol and all follow-up assessments. Statistical analyses will be conducted using SPSS version 22.0 (SPSS Inc., Chicago, IL, USA), with a significance level set at *P* < 0.05.

Demographic and clinical characteristics will be analyzed descriptively. Continuous variables will be presented as mean ± standard deviation, and categorical variables as frequencies and percentages. For between-group comparisons, continuous variables conforming to a normal distribution will be analyzed using independent samples t-tests, while non-normally distributed variables will be analyzed using rank-sum tests. Categorical variables will be compared using chi-square tests.

For the repeated measures data (including PDSS scale scores and PSG parameters) collected at baseline (Week 0), post-intervention (Week 4), and the end of the follow-up period (Week 6), the primary analysis will be performed using a linear mixed-effects model. The model will include group (REHAB vs. control), time point (as a categorical variable), and the group-by-time interaction term as fixed effects.

## 3 Discussion

The intervention approach designed in this study is heat therapy with REHAB. Heat therapy has recently emerged as a potential alternative to physical activity and exercise, inducing physiological stressors and subsequent positive adaptation mechanisms. Extensive cohort studies and reviews have highlighted the potential of heat therapy to improve physical and mental health for patients with cardiovascular disease (40), diabetes, peripheral arterial disease (41), arterial stiffness (42), glycemic control (43), and depression (44). Moreover, heat therapy may offer additional benefits in the context of neurodegenerative diseases concerning skeletal muscle function, cerebral blood flow, and metabolic health, including Alzheimer's disease (AD) and PD (17). Heat therapy is commonly administered through various methods such as hot bathing, hot water-perfused garments (45), heat pads (46, 47), heat chambers (48), hot sand baths (49, 50), and sauna bathing (51). At home, heat therapy is typically limited to hot bathing. REHAB is designed to enhance blood circulation by achieving a comfortably warm body temperature. It is as effective as full-body bathing but less physically demanding. This method is considered a safe, simple, and easily controllable approach (29).

There are currently no established guidelines regarding the clinical parameters of minimal heat therapy for improving health markers, including heating technique, duration, intensity, and timing. However, it is feasible to develop a protocol that is tolerable, effective, and enjoyable. In this study, key parameters such as temperature, duration, and frequency are clearly defined, enhancing the intervention's practicality and applicability.

Currently, there is no standardized temperature for hot water bathing. Most fundamental studies on thermotherapy aim to elevate core body temperature (CBT) to approximately 38.5 °C−39.0 °C; however, measuring CBT in clinical practice is challenging. Previous studies have used water temperatures ranging from 40 °C to 42 °C, with 41 °C being the most frequently reported in the literature (52–54). A comparative study found that only footbaths at 41 °C significantly increased CBT, whereas lower temperatures (e.g., 40 °C) only elevated the distal-to-proximal skin temperature gradient (DPG) (55). Similarly, Horne and Reid reported that a significant increase in pre-sleep fatigue—which may aid sleep onset—occurred only following a 41 °C bath (56). In addition, our preliminary patient survey indicated that this temperature offers the best balance between comfort and acceptability.

Considering previous findings that REHAB sessions exceeding 30 min can lead to discomfort and physiological strain, often resulting in pre-mature termination of treatment (57), we propose a protocol of 30-min sessions conducted 5–7 days per week for 4 weeks (25, 27). This strategy balances efficacy with participant comfort and feasibility.

The spectrum of types of sleep disturbance in PD patients are extensive and complex. Our study utilizes the PDSS as the primary outcome measure–a validated tool for assessing sleep in PD. This instrument evaluates multiple dimensions and symptom severity through a 15-item self-administered scale. Using this scale allows for a comprehensive assessment of sleep disorders, facilitates subgroup analysis in future research, and provides deeper insights into the mechanisms linking REHAB and sleep. In addition, our study incorporates PSG as an objective assessment tool, minimizing subjective bias in sleep evaluation. This approach enables precise observation of REHAB's impact on SWS and REM sleep, thereby enhancing research accuracy and robustness.

Importantly, hot bathing has long been utilized in medical practice as a therapeutic intervention (58). It offers the advantage of being easily implemented at home without requiring specialized equipment or professional supervision. Additionally, we provide clear guidelines regarding techniques, temperature, duration, and frequency to enhance both adherence and generalizability. This study contributes valuable insights into strategies for improving the quality of life for patients with advanced PD.

A primary limitation of this study stems from its open-label design. Firstly, due to the nature of the intervention (lower extremity hot water bathing), blinding of participants and treatment providers is not feasible, inevitably introducing the risk of performance bias. For instance, participants in the REHAB group, aware of receiving a novel intervention, may experience a stronger placebo effect due to positive outcome expectations or may more actively adhere to other sleep hygiene recommendations. Similarly, researchers may unintentionally devote more attention when guiding the REHAB group. Secondly, although we employed an outcome assessor-blinded design—where the neurologists responsible for collecting and analyzing polysomnography (PSG) data and assessing adverse events remain completely unaware of group allocation—the self-reported Parkinson's Disease Sleep Scale (PDSS), a subjective outcome, cannot avoid reporting bias introduced by the participants' own unblinded status. This constitutes one aspect of detection bias. By including objective PSG parameters as a core secondary endpoint, we aim to provide evidence for improvements in sleep architecture that is less influenced by subjective expectations, thereby partially mitigating this limitation. In future Phase III confirmatory trials, if feasible, consideration could be given to designing a “sham bathing” control (e.g., using water near skin temperature) to achieve participant blinding and more rigorously control for these biases.

Future research can build upon these findings by incorporating multicenter, large-sample designs. Such studies should examine the applicability of REHAB across various age groups, disease stages, and comorbidities, further expanding its clinical relevance and impact.

This study is designed to evaluate the safety and efficacy of REHAB as a rehabilitation intervention specifically for elderly patients with advanced PD who suffer from sleep disorders. The primary objective is to assess whether this therapy can be safely administered and whether it improves sleep quality in these patients. The experimental outcomes will help establish a novel rehabilitation strategy that could be integrated into guideline-based usual care for PD patients with sleep issues, especially for elderly patients with mobility limitations or other comorbidities. The results of this study will be instrumental in guiding future research and clinical practices.

## References

[1] Skogar Ö Nilsson M Lökk J. Gender differences in diagnostic tools, medication, time to medication, and nonmotor symptoms in Parkinsonian patients. Brain Circ. (2022) 8:192–9. doi: 10.4103/bc.bc_33_22 37181842 PMC10167852

[2] Stefani A Hogl B. Sleep in Parkinson's disease. Neuropsychopharmacology. (2020) 45:121–8. doi: 10.1038/s41386-019-0448-y 31234200 PMC6879568

[3] Iranzo A Cochen De Cock V Fantini ML Pérez-Carbonell L Trotti LM. Sleep and sleep disorders in people with Parkinson's disease. Lancet Neurol. (2024) 23:925–37. doi: 10.1016/S1474-4422(24)00170-4 38942041

[4] Taximaimaiti R Luo X Wang XP. Pharmacological and non-pharmacological treatments of sleep disorders in Parkinson's disease. Curr Neuropharmacol. (2021) 19:2233–49. doi: 10.2174/1570159X19666210517115706 33998990 PMC9185775

[5] Cristini J Weiss M De Las Heras B Medina-Rincón A Dagher A Postuma RB . The effects of exercise on sleep quality in persons with Parkinson's disease: a systematic review with meta-analysis. Sleep Med Rev. (2021) 55:101384. doi: 10.1016/j.smrv.2020.101384 32987321

[6] Amara AW Wood KH Joop A Memon RA Pilkington J Tuggle SC . Randomized, controlled trial of exercise on objective and subjective sleep in parkinson's disease. Mov Disord. (2020) 35:947–58. doi: 10.1002/mds.28009 32092190 PMC8826749

[7] Rutten S Vriend C Smit JH Berendse HW van Someren EJW Hoogendoorn AW . Bright light therapy for depression in Parkinson disease: a randomized controlled trial. Neurology. (2019) 92:e1145–e56. doi: 10.1212/WNL.0000000000007090 30770426

[8] Caparelli EC Abulseoud OA Gu H Zhai T Schleyer B Yang Y. Low frequency repetitive transcranial magnetic stimulation to the right dorsolateral prefrontal cortex engages thalamus, striatum, and the default mode network. Front Neurosci. (2022) 16:997259. doi: 10.3389/fnins.2022.997259 36248660 PMC9565480

[9] Zuzuárregui JRP Ostrem JL. The impact of deep brain stimulation on sleep in Parkinson's disease: an update. J Parkinsons Dis. (2020) 10:393–404. doi: 10.3233/JPD-191862 32250316 PMC7242854

[10] Kalaitzakis ME Gentleman SM Pearce RK. Disturbed sleep in Parkinson's disease: anatomical and pathological correlates. Neuropathol Appl Neurobiol. (2013) 39:644–53. doi: 10.1111/nan.12024 23363035

[11] Von Schulze AT Deng F Morris JK Geiger PC. Heat therapy: possible benefits for cognitive function and the aging brain. J Appl Physiol. (1985) 129:1468–76. doi: 10.1152/japplphysiol.00168.2020 32969779 PMC7792844

[12] Chauhan NR Kapoor M Prabha Singh L Gupta RK Chand Meena R Tulsawani R . Heat stress-induced neuroinflammation and aberration in monoamine levels in hypothalamus are associated with temperature dysregulation. Neuroscience. (2017) 358:79–92. doi: 10.1016/j.neuroscience.2017.06.023 28663093

[13] Novelli A Di Vico IA Terenzi F Sorbi S Ramat S. Dyskinesia-Hyperpyrexia Syndrome in Parkinson's disease with deep brain stimulation and high-dose levodopa/carbidopa and entacapone. Parkinsonism Relat Disord. (2019) 64:352–3. doi: 10.1016/j.parkreldis.2019.05.018 31101554

[14] Zhang P Huang P Li Y Du J Luo N He Y . Relationships Between Rapid Eye Movement sleep behavior disorder and Parkinson's disease: indication from gut microbiota alterations. Aging Dis. (2024) 15:357–68. doi: 10.14336/AD.2023.0518 37307829 PMC10796088

[15] Han J Zheng J Li Q Hong H Yao J Wang J . An antibody-directed and immune response modifier-augmented photothermal therapy strategy relieves aging via rapid immune clearance of senescent cells. Aging Dis. (2023) 15:787–803 doi: 10.14336/AD.2023.0628-1 38447216 PMC10917526

[16] Hunt AP Minett GM Gibson OR Kerr GK Stewart IB. Could heat therapy be an effective treatment for Alzheimer's and Parkinson's diseases? a narrative review. Front Physiol. (2019) 10:1556. doi: 10.3389/fphys.2019.01556 31998141 PMC6965159

[17] Heinonen I Laukkanen JA. Effects of heat and cold on health, with special reference to Finnish sauna bathing. Am J Physiol Regul Integr Comp Physiol. (2018) 314:R629–r38. doi: 10.1152/ajpregu.00115.2017 29351426

[18] Hussain JN Greaves RF Cohen MM. A hot topic for health: results of the global Sauna survey. Complement Ther Med. (2019) 44:223–34. doi: 10.1016/j.ctim.2019.03.012 31126560

[19] Lack LC Gradisar M Van Someren EJ Wright HR Lushington K. The relationship between insomnia and body temperatures. Sleep Med Rev. (2008) 12:307–17. doi: 10.1016/j.smrv.2008.02.003 18603220

[20] Ajwad A Huffman D Yaghouby F OrHara BF Sunderam S. Sleep depth enhancement through ambient temperature manipulation in mice. Annu Int Conf IEEE Eng Med Biol Soc. (2018) 2018:1392–5. doi: 10.1109/EMBC.2018.8512557 30440652

[21] Harding EC Franks NP Wisden W. The temperature dependence of sleep. Front Neurosci. (2019) 13:336. doi: 10.3389/fnins.2019.00336 31105512 PMC6491889

[22] Raymann RJ Swaab DF Van Someren EJ. Skin deep: enhanced sleep depth by cutaneous temperature manipulation. Brain. (2008) 131:500–13. doi: 10.1093/brain/awm315 18192289

[23] Sawatari H Nishizaka MK Miyazono M Ando SI Inoue S Takemoto M . Three nights leg thermal therapy could improve sleep quality in patients with chronic heart failure. Heart Vessels. (2018) 33:155–62. doi: 10.1007/s00380-017-1047-7 28905211

[24] Chiu HY Lin EY Chiu HT Chen PY A. Feasibility randomized controlled crossover trial of home-based warm footbath to improve sleep in the chronic phase of traumatic brain injury. J Neurosci Nurs. (2017) 49:380–5. doi: 10.1097/JNN.0000000000000325 29117034

[25] Yamamoto K Nagata S. Physiological and psychological evaluation of the wrapped warm footbath as a complementary nursing therapy to induce relaxation in hospitalized patients with incurable cancer: a pilot study. Cancer Nurs. (2011) 34:185–92. doi: 10.1097/NCC.0b013e3181fe4d2d 21252645

[26] Jafarimanesh H Vakilian K Mobasseri S. Thermo-therapy and cryotherapy to decrease the symptoms of restless leg syndrome during the pregnancy: a randomized clinical trial. Complement Ther Med. (2020) 50:102409. doi: 10.1016/j.ctim.2020.102409 32444058

[27] Park A Ambrogi K Hade EM. Randomized pilot trial for the efficacy of the MMF07 foot massager and heat therapy for restless legs syndrome. PLoS ONE. (2020) 15:e0230951. doi: 10.1371/journal.pone.0230951 32240228 PMC7117678

[28] Kjertakov M Petersen A. Hot water immersion could be an effective alternative to physical exercise in improving cardiovascular fitness during the COVID-19 pandemic. Front Physiol. (2022) 13:1035183. doi: 10.3389/fphys.2022.1035183 36505081 PMC9732457

[29] Turner B Pennefather J Edmonds C. Cardiovascular effects of hot water immersion (suicide soup). Med J Aust. (1980) 2:39–40. doi: 10.5694/j.1326-5377.1980.tb131813.x 7432266

[30] Chen Z Li G Liu J. Autonomic dysfunction in Parkinson's disease: implications for pathophysiology, diagnosis, and treatment. Neurobiol Dis. (2020) 134:104700. doi: 10.1016/j.nbd.2019.104700 31809788

[31] Antonini A Moro E Godeiro C Reichmann H. Medical and surgical management of advanced Parkinson's disease. Mov Disord. (2018) 33:900–8. doi: 10.1002/mds.27340 29570862

[32] Barki S Vibha D Pachipala S Tayade K Misra S Nath M . Safety and efficacy of fluoxetine in post-stroke anxiety: a pilot prospective randomized open blinded endpoint (PROBE) study. Int J Psychiatry Med. (2025) 60:495–507. doi: 10.1177/00912174241296233 39440836

[33] Fox SH Katzenschlager R Lim SY Barton B de Bie RMA Seppi K . International Parkinson and movement disorder society evidence-based medicine review: update on treatments for the motor symptoms of Parkinson's disease. Mov Disord. (2018) 33:1248–66. doi: 10.1002/mds.27372 29570866

[34] Seppi K Ray Chaudhuri K Coelho M Fox SH Katzenschlager R Perez Lloret S . Update on treatments for nonmotor symptoms of Parkinson's disease-an evidence-based medicine review. Mov Disord. (2019) 34:180–98. doi: 10.1002/mds.27602 30653247 PMC6916382

[35] Frazzitta G Maestri R Ferrazzoli D Riboldazzi G Bera R Fontanesi C . Multidisciplinary intensive rehabilitation treatment improves sleep quality in Parkinson's disease. J Clin Mov Disord. (2015) 2:11. doi: 10.1186/s40734-015-0020-9 26788347 PMC4711016

[36] Chaudhuri KR Pal S DiMarco A Whately-Smith C Bridgman K Mathew R . The Parkinson's disease sleep scale: a new instrument for assessing sleep and nocturnal disability in Parkinson's disease. J Neurol Neurosurg Psychiatry. (2002) 73:629–35. doi: 10.1136/jnnp.73.6.629 12438461 PMC1757333

[37] Kearney J Brittain JS. Sensory attenuation in sport and rehabilitation: perspective from research in Parkinson's disease. Brain Sci. 2021;11(5). doi: 10.3390/brainsci11050580 33946218 PMC8145846

[38] Hertzog MA. Considerations in determining sample size for pilot studies. Res Nurs Health. (2008) 31:180–91. doi: 10.1002/nur.20247 18183564

[39] Dobkin BH. Progressive staging of pilot studies to improve Phase III trials for motor interventions. Neurorehabil Neural Repair. (2009) 23:197–206. doi: 10.1177/1545968309331863 19240197 PMC4099048

[40] Brunt VE Minson CT. Heat therapy: mechanistic underpinnings and applications to cardiovascular health. J Appl Physiol. (1985) 130:1684–704. doi: 10.1152/japplphysiol.00141.2020 33792402 PMC8285605

[41] Akerman AP Thomas KN van Rij AM Body ED Alfadhel M Cotter JD. Heat therapy vs. supervised exercise therapy for peripheral arterial disease: a 12-wk randomized, controlled trial. Am J Physiol Heart Circ Physiol. (2019) 316:H1495–506. doi: 10.1152/ajpheart.00151.2019 31002283

[42] Brunt VE Eymann TM Francisco MA Howard MJ Minson CT. Passive heat therapy improves cutaneous microvascular function in sedentary humans via improved nitric oxide-dependent dilation. J Appl Physiol. (1985) 121:716–23. doi: 10.1152/japplphysiol.00424.2016 27418688 PMC6195670

[43] Maley MJ Hunt AP Stewart IB Faulkner SH Minett GM. Passive heating and glycaemic control in non-diabetic and diabetic individuals: a systematic review and meta-analysis. PLoS One. (2019) 14:e0214223. doi: 10.1371/journal.pone.0214223 30901372 PMC6430508

[44] Janssen CW Lowry CA Mehl MR Allen JJ Kelly KL Gartner DE . Whole-body hyperthermia for the treatment of major depressive disorder: a randomized clinical trial. JAMA Psychiatry. (2016) 73:789–95. doi: 10.1001/jamapsychiatry.2016.1031 27172277

[45] Kim K Monroe JC Gavin TP Roseguini BT. Skeletal muscle adaptations to heat therapy. J Appl Physiol. (1985) 128:1635–42. doi: 10.1152/japplphysiol.00061.2020 PMC7311689 32352340

[46] Goto K Oda H Kondo H Igaki M Suzuki A Tsuchiya S . Responses of muscle mass, strength and gene transcripts to long-term heat stress in healthy human subjects. Eur J Appl Physiol. (2011) 111:17–27. doi: 10.1007/s00421-010-1617-1 20803152

[47] Labidi M Ihsan M Behan FP Alhammoud M Smith T Mohamed M . Six weeks of localized heat therapy does not affect muscle mass, strength and contractile properties in healthy active humans. Eur J Appl Physiol. (2021) 121:573–82. doi: 10.1007/s00421-020-04545-9 33159573

[48] Ohno Y Egawa T Yokoyama S Nakai A Sugiura T Ohira Y . Deficiency of heat shock transcription factor 1 suppresses heat stress-associated increase in slow soleus muscle mass of mice. Acta Physiol (Oxf). (2015) 215:191–203. doi: 10.1111/apha.12600 26347147

[49] Ihsan M Deldicque L Molphy J Britto F Cherif A Racinais S. Skeletal Muscle Signaling Following Whole-Body and Localized Heat Exposure in Humans. Front Physiol. (2020) 11:839. doi: 10.3389/fphys.2020.00839 32765299 PMC7381176

[50] Antonelli M Donelli D. Hot sand baths (psammotherapy): a systematic review. Complement Ther Med. (2019) 42:1–6. doi: 10.1016/j.ctim.2018.10.020 30670225

[51] Toro V Siquier-Coll J Bartolomé I Pérez-Quintero M Raimundo A Muñoz D . Effects of twelve sessions of high-temperature sauna baths on body composition in healthy young men. Int J Environ Res Public Health. (2021) 18:4458. doi: 10.3390/ijerph18094458 33922289 PMC8122786

[52] Gerrett N Alkemade P Daanen H. Heat reacclimation using exercise or hot water immersion. Med Sci Sports Exerc. (2021) 53:1517–28. doi: 10.1249/MSS.0000000000002612 34127636 PMC8208095

[53] Greenfield AM Pereira FG Boyer WR Apkarian MR Kuennen MR Gillum TL. Short-term hot water immersion results in substantial thermal strain and partial heat acclimation; comparisons with heat-exercise exposures. J Therm Biol. (2021) 97:102898. doi: 10.1016/j.jtherbio.2021.102898 33863451

[54] Haghayegh S Khoshnevis S Smolensky MH Diller KR Castriotta RJ. Before-bedtime passive body heating by warm shower or bath to improve sleep: a systematic review and meta-analysis. Sleep Med Rev. (2019) 46:124–35. doi: 10.1016/j.smrv.2019.04.008 31102877

[55] Liao WC Landis CA Lentz MJ Chiu MJ. Effect of foot bathing on distal-proximal skin temperature gradient in elders. Int J Nurs Stud. (2005) 42:717–22. doi: 10.1016/j.ijnurstu.2004.11.011 16084919

[56] Horne JA Reid AJ. Night-time sleep EEG changes following body heating in a warm bath. Electroencephalogr Clin Neurophysiol. (1985) 60:154–7. doi: 10.1016/0013-4694(85)90022-7 2578367

[57] Hoekstra SP Bishop NC Faulkner SH Bailey SJ Leicht CA. Acute and chronic effects of hot water immersion on inflammation and metabolism in sedentary, overweight adults. J Appl Physiol. (1985) 125:2008–18. doi: 10.1152/japplphysiol.00407.2018 30335579

[58] Naumann J Grebe J Kaifel S Weinert T Sadaghiani C Huber R. Effects of hyperthermic baths on depression, sleep and heart rate variability in patients with depressive disorder: a randomized clinical pilot trial. BMC Complement Altern Med. (2017) 17:172. doi: 10.1186/s12906-017-1676-5 28351399 PMC5371197

